# SAVE-CLC: An Intervention to Reduce Suicide Risk in Veterans Who Discharge From VA Nursing Homes

**DOI:** 10.1093/geroni/igaa057.302

**Published:** 2020-12-16

**Authors:** Kelsey Simons, Katherine Luci, Lauren Hagemann, Lindsey Jacobs, Emily Bower, Morgan Eichorst, Michelle Hilgeman

**Affiliations:** 1 VA Medical Center--Canandaigua, Canandaigua, New York, United States; 2 Salem VA Medical Center, Salem, Virginia, United States; 3 Salem VAMC, Salem, Virginia, United States; 4 VA Boston Healthcare System, Brockton, Massachusetts, United States; 5 Veterans Health Administration, Canandaigua, New York, United States; 6 VA Northern Indiana, Mishawaka, Indiana, United States; 7 VA Medical Center--Tuscaloosa, Tuscaloosa, Alabama, United States

## Abstract

Prior research has established transitions into and out of nursing homes as periods of suicide risk for older adults. Deaths by suicide were found to be 2.4 times as likely among Veterans within six months of discharge from US Veterans Health Administration (VA) nursing homes when compared with gender and age-matched Veterans from the general VA patient population (McCarthy, Szymanski, Karlin, & Katz, 2013). Despite these trends, suicide prevention interventions implemented during nursing home and post-acute care transitions, including those taking place from Centers for Medicare and Medicaid Services regulated nursing homes, are lacking. Suicide Awareness for Veterans Exiting the Community Living Center (SAVE-CLC) was piloted as a quality improvement intervention to reduce suicide risk for older Veterans discharging from VA nursing homes. VA clinicians from three sites provided a friendly contact by phone after discharge (n = 66) to screen for depression, facilitate a strengths-based discussion about service needs, and provide service referrals. Compared to a group of patients discharged prior to the start of the intervention (matched on location, age range, and Care Assessment Need scores), SAVE-CLC patients received more depression screening within 30 days after discharge (chi square = 38.7, p < .001) and were seen more quickly for mental health care (t = 3.1, p = .005) when indicated. Implications for suicide prevention with older Veterans and for the general population of older adults receiving short stay services in US nursing homes will be addressed.

